# Illness risk representations underlying women's breast cancer risk appraisals: A theory‐informed qualitative analysis

**DOI:** 10.1111/bjhp.12792

**Published:** 2025-03-13

**Authors:** Victoria G. Woof, Lorna McWilliams, D. Gareth Evans, Anthony Howell, David P. French

**Affiliations:** ^1^ University of Manchester Manchester UK; ^2^ The Nightingale Centre, Wythenshawe Hospital, Manchester University NHS Foundation Trust Manchester UK

**Keywords:** breast cancer, framework analysis, illness representations, illness risk representation framework, qualitative analysis, risk appraisals

## Abstract

**Objectives:**

This study assessed the utility of Cameron's Illness Risk Representation (IRR) framework in understanding how women interpret their breast cancer risk after receiving a clinically derived estimate.

**Design:**

Secondary qualitative analysis of two studies within the BC‐Predict trial, using semi‐structured telephone interviews with women aged 47–74 who received breast cancer risk estimates via population screening.

**Methods:**

Forty‐eight women were informed of their 10‐year breast cancer risk (low (<1.5% risk), average (1.5–4.99%), above‐average (moderate; 5–7.99%) and high (≥8%)). Moderate‐ and high‐risk women were eligible for enhanced preventive management. Women were interviewed about their risk, with data analysed using a thematic framework approach.

**Results:**

Causal representations of breast cancer were often incomplete, with women primarily relying on family history and health‐related behaviours to understand their risk. This reliance shaped pre‐existing expectations and led to uncertainty about unfamiliar risk factors. As women aged, concerns about breast cancer susceptibility became more prominent. Emotional reactions to risk communication, along with the physical implications of risk management strategies, were also considered. Women were knowledgeable about early detection and prevention strategies, showing agency in reducing risk and preventing aggressive cancers.

**Conclusions:**

The IRR framework largely explained women's breast cancer risk appraisals but adaptations could enhance its applicability. The identity construct could be redefined and combined with the causal construct. The framework should also consider the extent to which pre‐existing appraisals change after receiving a clinical‐derived risk estimate. Healthcare professionals should assess women's knowledge before communicating personal risk estimates to reduce doubt and the impact of unfamiliar information.

**Statement of contribution**

**What is already known on this subject?**

- Women's personal appraisals of their breast cancer risk do not always reflect the objective risk estimates they have been provided with, but it is unclear why.
- Cameron's Illness Risk Representation (IRR) framework has been used to explain personal risk appraisals and preventative decision‐making in relation to other diseases.

**What does this study add?**

- There are gaps in women's knowledge about the risk factors and causes of breast cancer.
- The IRR framework can explain breast cancer risk appraisals but adaptations are needed in this context.
- The framework could inform interventions to alter risk appraisals, enabling informed preventative decisions.

## BACKGROUND

Healthcare professionals (HCPs) frequently estimate and communicate an individual's risk of illness, particularly for diseases like cardiovascular disease (CVD), diabetes and breast cancer, by measuring various associated risk factors. For breast cancer, the risk of disease can be predicted by factors including age, family history, hormonal and reproductive factors (e.g., age at menarche and parity) and health behaviours. If deemed suitable, HCPs may recommend preventive measures to reduce the risk of breast cancer, that is additional screening, preventative medication and health behaviour adjustments (National Institute for Health and Care Excellence (NICE), 2013).

A challenge which has previously been acknowledged when communicating breast cancer risk is that women tend to hold inaccurate risk appraisals. For instance, even after a clinical risk estimate has been communicated, women's personal risk appraisals still do not fully align with the estimate provided, with recall accuracy only improving modestly (Bayne et al., 2020; Cull et al., 1999; Lobb et al., 2003; Ozanne et al., 2010). A recent review of the qualitative research available in this area revealed that women hold misperceptions regarding their risk of breast cancer, with personal risk appraisals largely influenced by the presence or absence of a family history (Woof et al., 2022). Additionally, it has been found that women in receipt of an increased 10‐year breast cancer risk estimate (communicated via a letter and calculated using family history data, hormonal and reproductive data and health behaviour information) from population breast screening (a public health programme that routinely invites women to be screened based on age (in the UK between 50 and 70) for the purpose of detecting breast cancer early) do not always personally identify or agree with the clinical risk estimate provided, citing that their family history and health behaviours do not match with their understanding of the causes of breast cancer or of being at increased risk (Woof, McWilliams, et al., 2023). Holding such inaccurate or incomplete appraisals of risk can significantly affect views on preventative management. For instance, those who underestimate their risk may not take up beneficial preventive options, such as preventative medication or health behaviour changes, thereby missing opportunities to reduce their risk. Facilitating the formation of accurate risk appraisals is therefore essential for aiding women's informed decisions about preventive management options.

Although a small body of research exists which attempts to explore how women's personal breast cancer risk appraisals are formed, it is not clear what specific beliefs and factors women rely on to formulate these appraisals. Drawing on the principles of Leventhal's Common Sense Model of Illness Self‐Regulation (CSM‐SR) (Leventhal et al., 2012, 2013), Cameron's Illness Risk Representation (IRR) framework attempts to provide a theoretical understanding of how representations of risk and personal risk appraisals develop and how these appraisals influence preventative behaviour (Cameron, 2003, 2008). Cameron's IRR framework applies the five cognitive constructs of self‐regulation outlined in the CSM‐SR that have been suggested to directly influence constructs related to the risk appraisal literature, including estimates of probability (likelihood) and evaluations of severity (Cameron, 2003, 2008). These cognitive constructs include: *identity* (the description of the illness, including symptoms), *cause* (the causal factors associated with the illness), *timeline* (how long the illness lasts and the point of onset), *consequences* (the social, psychological and physical impact of the illness) and *controllability* (agency over the illness and beliefs about curability) (Cameron, 2003, 2008). It is suggested by Cameron that components, *identity, timeline* and *cause* are associated with likelihood estimates, with *consequences* and *controllability* feeding into severity estimates. Cameron also proposes that emotional responses to an illness threat can influence illness representations and risk appraisals, independent of cognitive reasoning (Cameron, 2003).

According to Cameron (Cameron, 2003), evaluations of an illness risk are not just shaped by an individual's perception of the illness itself. These assessments are dependent on aligning personal attributes with one's own conceptualization of the illness (Cameron, 2003). For instance, a woman's perception of her risk of breast cancer may stem from relating personal characteristics (such as possessing a family history) with the factors she believes contribute to breast cancer, influencing her future coping strategies (e.g., considering preventive medication). However, there may be discrepancies between an individual's self‐perception of risk and their conceptualization of the illness (Cameron, 2003). For example, a woman might acknowledge that she is overweight, yet fail to recognize this as a contributing factor for breast cancer. Consequently, she may not adopt preventive behaviours to mitigate her risk. In this scenario, her appraisal of her risk could be viewed as incomplete or deficient in understanding the causal factors related to breast cancer.

Qualitative evidence for the utility of the IRR framework has been found in other cancers and disease areas, including for CVD (Newby et al., 2020) and bowel cancer (Newby et al., 2017). The qualitative methods employed in these previous studies facilitated a thorough examination of the IRR framework constructs, while also providing the flexibility to identify risk representation areas which may not have been fully explained by the framework. Through these qualitative methods, these studies found that the IRR framework largely captured risk beliefs. However, a key limitation is that participants did not receive personal risk estimates prior to being interviewed, so the studies examined relatively static beliefs and not how these beliefs are affected by the provision of new information. Thus, it is currently unknown how useful the IRR framework is in the context of breast cancer, as well as in understanding risk representations following the communication of a clinically derived risk estimate.

Given the frequent misalignment between clinically communicated breast cancer risk estimates and women's breast cancer risk appraisals (Cull et al., 1999; Lobb et al., 2003; Ozanne et al., 2010; Woof, McWilliams, et al., 2023), there is an urgent need to understand how women form personal appraisals of their risk, including the beliefs and factors influencing these perceptions. Such an understanding is timely, as the communication of breast cancer risk in clinical settings is increasingly being carried out, and may soon become routine practice at population breast screening (McWilliams et al., 2022). Such an understanding could inform interventions aimed at improving the accuracy of risk appraisals, thereby facilitating informed decisions regarding preventive management and health behaviour changes. The present study therefore seeks to investigate how useful Cameron's IRR framework (Cameron, 2003, 2008) is in understanding how women, upon receiving a clinically derived breast cancer risk estimate, make sense of their risk and what informs their risk representations.

## METHODS

### Design

The datasets for the secondary analysis presented here have been taken from two qualitative studies (Woof, McWilliams, et al., 2023; McWilliams et al., 2023; hereafter known as study one and study two) nested within the Breast Cancer Predict (BC‐Predict) trial, where women received 10‐year personalized breast cancer risk estimates as part of population breast screening. Semi‐structured one‐to‐one telephone interviews were conducted with women in each of these studies, following receipt of risk estimates.

### Setting and participants

The BC‐Predict study (Evans et al., 2023; French et al., 2020) aimed to assess the feasibility of implementing breast cancer risk estimation and stratification as part of the NHS Breast Screening Programme (NHSBSP). This feasibility study was conducted in the North West of England across seven breast screening sites run by three NHS Trusts. To be eligible to participate, women were required to be of screening age and born biologically female [see (French et al., 2020) and (Evans et al., 2023) for full eligibility criteria].

Eligibility for study one (Woof, McWilliams, et al., 2023) included women who participated in the BC‐Predict study who had been notified of an above‐average (moderate) or high 10‐year breast cancer risk estimate and whose risk estimates included a polygenic risk score (a calculation of genetic variants (single nucleotide polymorphisms (SNPs)) related to hereditary breast cancer). Eligibility for study two (McWilliams et al., 2023) included women who had been notified of a low, average, above‐average (moderate) or high 10‐year breast cancer risk estimate from the BC‐Predict study. Women in the BC‐Predict study were given breast cancer risk estimates based on the following categories outlined in NICE clinical guidelines: low (<1.5% risk), average (1.5–4.99%), above‐average (moderate; 5–7.99%) and high (≥8%) (NICE, 2013; 2019). Alongside health behaviour advice, more frequent screening (every 12–18 months) and preventative medication were offered to those found to be at above‐average (moderate) and high risk of breast cancer in the BC‐Predict study. Women at average and low risk were also offered health behaviour advice and to continue with 3‐yearly screening in line with the NHSBSP.

Interviews in study one aimed to explore, in‐depth, how those women who had received an increased risk estimate (above‐average [moderate] or high) understood and made sense of breast cancer and their risk of developing the disease (Woof, McWilliams, et al., 2023). Interviews conducted in study two predominantly focused on how women from low, average, above‐average (moderate) and high‐risk groups viewed their participation in the BC‐Predict study, as well as their understanding of their breast cancer risk (McWilliams et al., 2023). By analysing these two datasets together, the secondary analysis presented here aimed to assess the utility of Cameron's IRR framework (Cameron, 2003, 2008) in understanding how women interpret a clinically derived breast cancer risk estimate and what influences their risk representations/appraisals.

### Procedure and materials of primary studies

In the BC‐Predict study, women first received their invitation for 3‐yearly breast screening from the NHSBSP before information was distributed about the study. Those interested in participating were required to fill in a self‐report questionnaire asking for details on their family history, hormonal and reproductive factors (e.g., age at menarche and parity) and health behaviours. Breast density data from mammogram images and, for a subset of women, a polygenic risk score derived from saliva samples were combined with self‐report information to calculate a personalized 10‐year breast cancer risk estimate. Following the communication of a clear screening result, women received their estimated 10‐year breast cancer risk via postal letter [see (Evans et al., 2023; French et al., 2020) for a more detailed procedure of the BC‐Predict study and Supplementary Material S1 for BC‐Predict risk communication materials].

Following this, women in studies one and two (McWilliams et al., 2023; Woof, McWilliams, et al., 2023) were invited to participate in an interview via invite letters and participant information sheets administered by post by the BC‐Predict study team. In both studies, women at above‐average (moderate) and high risk were invited for an interview at least 6 months after they had received their risk feedback. This was so that any content discussed in the interviews would not influence decisions as to whether to attend a risk consultation to discuss their risk feedback and preventative management options. Women in study two (McWilliams et al., 2023) at low or average risk were invited to interview approximately 4 weeks after receiving their risk feedback.

Ethical approval for both studies was granted by the North West—Greater Manchester East Research Ethics Committee (18/NW/0856). For each study, topic guides were designed. For study one (Woof, McWilliams, et al., 2023) the topic guide focused on women's experiences and understandings of breast cancer, breast cancer risk in general and thoughts on their personal risk of developing the disease (see Supplementary Material S1). For study two (McWilliams et al., 2023), the topic guide comprised of questions relating to women's involvement in the BC‐Predict study and their thoughts regarding the introduction of risk stratified screening at the population level. Topic guides in both studies were informed by a Public Patient Involvement and Engagement (PPIE) group, who had experience of receiving breast cancer risk feedback (Supplementary Material S1).

### Suitability for secondary data analysis

When deciding whether to conduct a secondary analysis of qualitative data, it is important to consider the following: (i) the quality and the completeness of the original data, (ii) the fit of the original data with the new research question(s), (iii) the potential for new insights, (iv) the context in which the original dataset(s) were collected and (v) ethical restrictions (Heaton, 2008; Ruggiano & Perry, 2019).

When assessing the suitability of the datasets for secondary data analysis, the primary author read several transcripts from each set. It was evident from reading transcripts from study one (Woof, McWilliams, et al., 2023) that the data were sufficient to answer the present research question. Indeed, it was this study on primary analysis that the primary author identified evidence of the IRR framework, which prompted this secondary analysis. Although the data generated from study two (McWilliams et al., 2023) did not align as closely with the present research question, the primary author concluded that there was sufficient depth of data pertaining to breast cancer risk appraisals, making this dataset appropriate for a secondary analysis. Specifically, data regarding breast cancer risk appraisals in study two (McWilliams et al., 2023) warranted further consideration, as these data were not the focus of the primary analysis. And so the authors believed that the datasets had the potential to generate new insights which could answer the present research question and add to the wider literature on breast cancer risk appraisals and illness risk representations.

With regards to the context in which the data for study one (Woof, McWilliams, et al., 2023) and two (McWilliams et al., 2023) were collected, both studies and the data generated aligned well, as participants in each study took part in the BC‐Predict feasibility trial and the methods used for data collection and analysis were compatible for a secondary data analysis. Finally, both studies received consent from participants to share their study data with other researchers for the purpose of future research, making a secondary data analysis possible.

### Secondary data analysis

Original data from study one were analysed using interpretive phenomenological analysis (IPA). Data from study two were analysed using reflexive thematic analysis. Details of the analysis process can be found in the corresponding published papers (McWilliams et al., 2023; Woof, McWilliams, et al., 2023).

The secondary qualitative analysis presented here aimed to assess the utility of the IRR Framework (Cameron, 2003, 2008) for understanding women's breast cancer risk appraisals following the communication of a clinically derived risk estimate from the BC‐Predict study, which aimed to provide women with their 10‐year risk of developing breast cancer as part of population screening. Data were analysed in NVivo12 using thematic analysis and managed using a framework approach from a critical realist perspective (Brooks & King, 2017; Gale et al., 2013; Ritchie & Spencer, 2002). As this study aimed to assess the utility of Cameron's IRR framework (Cameron, 2003, 2008), this approach was chosen as it allows for both a deductive analysis based on existing theory, as well as enabling flexibility to develop inductive codes which may not be captured under an existing deductive framework. Furthermore, as both study one (Woof, McWilliams, et al., 2023) and study two (McWilliams et al., 2023) employed interpretive methods of data analysis (IPA and reflexive thematic analysis), a framework approach enabled flexibility to account for these two analysis methods while also applying structure to data that was more interpretive in nature (Heaton, 2008; Ruggiano & Perry, 2019). Using framework analysis in this way allowed the primary author to build on the original interpretations while adding new layers of organization and understanding (Heaton, 2008; Ruggiano & Perry, 2019).

Primary data analysis was conducted by VGW, with input from DPF. The analysis process began with familiarization of the transcripts, with VGW noting down any initial impressions of the data. Following the coding of 4 transcripts (1 from each risk group), an initial set of codes was developed which captured the main 5 components of the IRR framework. For example, for ‘*consequences*’, codes were developed to capture thoughts relating to the physical, psychological and social aspects of being at risk or being diagnosed with breast cancer. This initial coding framework was then used to code further transcripts. Inductive coding was utilized and additional codes added if the existing framework did not capture important features of the interview. Following the analysis of 12 transcripts (3 from each risk group), the framework categories were refined and a working framework agreed upon by VGW and DPF (see Supplementary Material S1). This framework was then applied to subsequent interview transcripts. Framework categories were then organized into separate matrices using the ‘framework’ feature in NVivo12 and exported to Microsoft Excel. Within these individual matrices, data were summarized per participant (case) and illustrative quotes provided. VGW then interpreted the data and developed analytical themes based on the IRR framework and additional inductive codes. These themes were then refined (VGW & DPF) and the final analysis agreed upon.

Due to technical difficulties in study two, one interview could not be transcribed accurately. Instead, comprehensive notes were taken by the researcher at the time and integrated into this secondary framework analysis to assess whether this woman's account of receiving a breast cancer risk estimate differed significantly from the opinions expressed in the wider dataset.

## RESULTS

Across both studies (McWilliams et al., 2023; Woof, McWilliams, et al., 2023), forty‐eight women were interviewed. Table 1 shows the characteristics of the sample. The final framework analysis results in four themes, with theme one having two subthemes: (i) The interplay between identity and cause in forming risk appraisals, (ii) Timeline—aging and the relevance of breast cancer, (iii) Consequences—social and prevention management consequences and (iv) Control—affective regulation and agency over prevention. All quotes are anonymized, and the same pseudonyms employed as in the primary studies.

**TABLE 1:** Characteristics of women in the sample.

Characteristics	Number of women (n)		
	Study one (anonymized)	Study two (anonymized)	Overall total from studies one & two
Risk group			
Low	–	10	10
Average	–	9	9
Moderate (above‐average)	7	11	18
High	1	10	11
Age			
47–54 years	2	13	15
55–64 years	6	26	32
65–74 years	–	1	1
IMD decile^a^			
1	–	1	1
2	–	2	2
3	–	–	0
4	1	5	6
5	–	1	1
6	–	4	4
7	1	2	3
8	5	5	10
9	1	7	8
10	–	13	13
Ethnicity			
Black African or Caribbean	–	1	1
White British or Irish	8	36	44
Other	–	1	1
Unknown	–	2	2

^a^ Index of multiple deprivation; higher number indicates least deprived (Ministry of Housing, Communities, Local Government, 2019).

### Theme 1: The interplay between identity and cause in forming risk appraisals

This theme illustrates the interconnected nature of the IRR framework's constructs, *identity* and *cause* in the context of forming breast cancer risk appraisals. These constructs reveal how women's perceptions of their susceptibility to breast cancer are shaped by family history and health behaviours. As many frame the disease in this way, clinical risk estimates often challenge these beliefs, leading to surprises or doubts when risk categories do not align with personal expectations. This tension highlights the complexity of integrating new risk information with existing views on prevention and risk management.

#### Subtheme 1a: Breast cancer, a hereditary disease

Although many of the women considered behaviour‐related risk factors (i.e., diet and exercise) when defining breast cancer, ultimately breast cancer was characterized as a genetic or hereditary disease:

Cause it can tend to be a family thing, can’t it? It can be, like, run through the generations. (Yvonne, moderate risk, study 1)

As interviews focused primarily on the communication of a breast cancer risk, women largely reflected on how they viewed breast cancer risk categories. Low‐ and average‐risk women identified that a lack of family history and positive health behaviours (i.e., being a healthy weight, not smoking and exercising) were the predominant risk factors influencing these estimates. Specifically, it seemed important to define these categories as, *low risk but not no risk*. These women were conscious not to define themselves as immune to the development of breast cancer, with attending screening and being cognisant of the small likelihood of a diagnosis remaining important:

…breast cancer can happen to anybody, so even if I'm in a low category the chance is not zero, so in five or four years' time, yes, it might still have happened. So I'm not ecstatic, saying it's never going to happen to me, I'm happy the category is low but I do realise that it is not an absolute. (Sharon, low risk, study 2).

Those at moderate and high risk also viewed the presence of a family history and unhealthy behaviours as significant causes and features of an increased risk estimate. Very few women were previously aware of the hormonal and reproductive risk factors associated with the development of breast cancer, aside from HRT use. In particular, risk estimates that took into account parity and having children over the age of 30 years were new risk factors that women had not previously considered when reflecting on their likelihood of developing the disease.

Symptoms, such as lumps, breast changes and breast cancer affecting men as well as women, were also briefly considered when describing breast cancer, with some women explaining that they had sought medical advice and had lumps examined to rule out the presence of breast cancer. However, one woman at low risk explained that even though pain under the armpit could be indicative of breast cancer, she would not seek medical advice, instead attributing this pain to carrying heavy bags regularly.

#### Subtheme 1b: Changes in causal representations—The stability of pre‐existing expectations

For many, the communication of a clinically derived breast cancer risk estimate challenged pre‐existing expectations of risk. As highlighted in *subtheme 1a*, family history and health behaviours appeared to dominate women's expectations and appraisals of their risk, using these appraisals to make assumptions about their risk results prior to receiving feedback:

I can't see why I would be any higher risk than anybody else, because I know there's no family medical history of breast cancer, I'm not overweight, I don't smoke, I exercise, I eat well… (Rachel, average risk, study 2).

Specifically, it was clear from the interviews that the presence or absence of a family history was a significant factor for women when evaluating the likelihood of developing the disease, with some women disbelieving or feeling dubious about their risk estimates due to the stability of these causal representations of breast cancer:

I was surprised at my risk category when I got the letter from the Predict study, that I'm in low risk because of my family history of cancer. I was surprised and I don't know, I almost perhaps don't believe that even though it says I'm in the lowest risk ‘cause I think because of having the family history, I tend to think I’m a lot more at risk. So it was good news to get the low risk, but I don't know whether I'm convinced by it almost. (Hannah, low risk, study 2).

Seemingly stable views about breast cancer being caused by a family history and unhealthy behaviours meant that risk results were unexpected and worrying for some. For instance, when these particular risk factors did not inform moderate or high‐risk estimates, some women appeared surprised as their causal representations of breast cancer were incomplete:

I thought I'm going to be fine here ‘cause I'm, you know, I'm fit and healthy, I do a lot of exercise, I'm not overweight, I don't drink a lot, eat healthy food, no history of cancer in our family, and I came back as high risk. And it turns out it's because I haven’t had children and I went into the menopause late. (Olive, high risk, study 2).

From Olive, however, we do see an acknowledgment of new risk factors and the influence they have had on her risk. Nevertheless, although the majority of women seemed to accept their clinically derived risk estimates, study two revealed that some women struggled to reconcile these estimates with their pre‐existing expectations and representations of their risk. As a result, some did not seem to internalize this information or view themselves as candidates for preventive management, especially for preventive medication.

### Theme 2: Timeline—Aging and the relevance of breast cancer

Most women identified that typically breast cancer is described as an older women's disease. One woman expressed that this was her belief when she was younger, identifying that she never worried about developing breast cancer during this time. However, women drew on instances where they had seen younger women diagnosed with breast cancer in their personal lives or in the media to conclude that the disease can affect women at any life stage. For women who had experienced breast cancer in the family or in a close friend, evaluating the age of onset against their own age increased the relevance of the disease to them. Approaching the age at which significant others were diagnosed or died from the disease was a fearful or nervous time for some and increased their awareness of their chances of developing breast cancer. However, aging passed this point helped to alleviate the worry associated with the likelihood of a diagnosis:

…she [participant's mother] had another scare a few years later so I grew up thinking that I would never see 40, absolutely convinced of it. And as I've gone past my 40s and I'm now in my 60s I'm not quite as obsessive about it but it's played a huge, a huge part of my life… (Jennifer, moderate risk, study 2).

Women also acknowledged becoming more conscious of their health with age. Specifically, seeing women of a similar age with breast cancer or other illnesses influenced their thoughts on their risk and the relevance of the disease to them:

So, at my age now, I know people, friends or colleagues that have had breast cancer, so it becomes more applicable to me. (Liz, low risk, study 2).

…my friend who was diagnosed with it, it was just, yeah, thinking about it more than I have done in the past, shall we say? (Sue, moderate risk, study 1).

### Theme 3: Consequences—Social and prevention management consequences

As interviews primarily focused on receiving a breast cancer risk estimate, women did not focus too closely on the consequences of a breast cancer diagnosis. Instead, women spoke about the consequences of receiving a personal risk estimate. For example, some women considered the consequences of their risk status with regard to their relationships with others. There was acknowledgment that the communication of a personal risk estimate does not just affect the recipient but has consequences for others within the family, be that increasing the awareness of breast cancer among female relatives or triggering worry and concern. For example, some women chose to keep their risk estimates to themselves to prevent causing worry and anxiety in close female relatives and children:

So, my sister is always messaging me, oh she said I hate this, ‘cause it's like one in two, so I didn’t want to tell her ‘Cause I thought, if I tell her I'm high risk she'll be worried, and I don't want her to worry. (Olive, high risk, study 2).

And I have to say that for days and days afterwards, I couldn't sleep, I couldn't think about anything else. I kept thinking about how I’m going to tell my son… (Sue, moderate risk, study 1).

Rather than consider the consequences of a diagnosis, communication of a clinical risk estimate generated thoughts on the consequences of different early detection and preventative management options. Although the majority of women expressed the importance of attending for regular breast screening, some acknowledged the health implications of an increased exposure to radiation if advised to attend more frequently. However, the majority of women at moderate and high risk who raised this issue considered the harms in relation to the perceived benefits of additional screening, arguing that for them increased surveillance, reduced worry and achieving peace of mind outweighed this risk:

I knew that there was a slight downside to that because you shouldn’t be exposing yourself to x‐ray or whatever the radiography risk is. But it seemed to me that that was a price worth paying just to get a peace of mind that there was nothing too worrying each year. (Gemma, high risk, study 2).

In addition to more frequent screening, women at moderate and high risk are also considered for and offered preventative medication. Although women understood that taking this medication would reduce the likelihood of a diagnosis, many women at moderate and high risk were opposed, concerned about any potential side effects which could disrupt their lives. One woman in particular drew on her experiences of witnessing her Auntie's use of tamoxifen (a preventive medication), which appeared to reduce her quality of life, informing her decision not to take preventive medication to reduce her risk:

I felt on reflection that I probably wouldn't go down that route simply because I think it's the drug that my aunt's been on and she said that she had had some horrendous side effects with it. (Jennifer, moderate risk, study 2).

### Theme 4: Control—Affective regulation and agency over prevention

There was a general awareness from the women that breast cancer is perhaps one of the most treatable cancers if caught early, with treatments being effective and survival likely. Due to this, women did not seem to be too fearful of a diagnosis, as hopefully by attending screening, being breast aware and remaining healthy, any cancers diagnosed would be caught early. Thus, the majority of women highlighted the importance of early detection to reduce the severity of breast cancer if diagnosed and saw breast screening as the ‘gold standard’ for achieving this. Women explained having agency over their attendance and viewed screening as giving them the best chance of reducing the likelihood of developing an aggressive cancer. The majority of women did not consider their breast cancer risk estimates in relation to their decisions to attend for screening, instead explaining that attendance was crucial no matter what level of risk they possessed:

Yes, my results came back very low, however, I'm quite keen to keep being checked very regularly so although I might be low risk…I think it's every three years… And I like that, from my point of view I'd rather it was kept at that. (Tina, low risk, study 2).

Together with the motivation for detecting cancers at an earlier stage, women were also motivated to attend screening due to the reassurance and peace of mind it affords them following a negative result, reducing breast cancer‐related anxiety. To aid early detection further, the majority of women explained engaging in breast self‐examinations. For those who shared that they did not check their breasts as often as they felt they should, communication of a breast cancer risk estimate made them more conscious to do so. Many of the women identified that checking their breasts, be that regularly or more sporadically provided them with the opportunity to seek medical advice if any changes were felt:

I also do the, you know, self‐examinations and things like that so if I thought that something had changed and I was concerned about something then I would contact my doctor or whatever. So I'm quite fastidious about that. (Abigail, average risk, study 2).

Together with attending for breast screening and engaging in breast self‐examinations, women also identified the agency they possessed over their health behaviours to either maintain or reduce their risk of developing breast cancer. The majority of women explained that they felt that they were doing all they could already to mitigate their risk, by eating healthily, engaging in exercise, not smoking and limiting alcohol intake. Additionally, women claimed that engaging in positive health behaviours benefits their overall health in general and enacting these behaviours was not solely linked to reducing the likelihood of developing breast cancer:

I am fairly fit. I don't eat processed foods, I cook everything. I probably do eat too much dairy, and drink too much wine. But other than that, you know, yes, it's all sort of across the board for anything, isn't it really? It's not just breast cancer, it's kinda like you just know what you should be doing. (Mina, high risk, study 2).

For some women, the use of preventative medication to reduce risk was not something they had previously had an awareness of. Although a small minority who were eligible opted to reduce their risk in this way, many felt they needed a deeper understanding of the medication before taking it. Yet most of these women believed that the risks of the medication outweighed the benefits of reducing the likelihood of developing breast cancer. Overall, women demonstrated a significant level of knowledge regarding the preventative management options available to reduce their risk and the likelihood of developing an aggressive cancer.

## DISCUSSION

This study demonstrated that women's appraisals of their breast cancer risk are primarily informed by the presence or absence of a family history and an evaluation of health behaviours. How women reacted to and understood their clinically derived risk estimates was affected by how stable these causal representations were. There were limitations in knowledge of other breast cancer risk factors, such as parity and age at first pregnancy before receiving a breast cancer risk estimate, leading some women to question the risk estimates they received. Aging increased the relevance of breast cancer for women, with women identifying that the chances of developing breast cancer increase with age. However, women did acknowledge that breast cancer can be diagnosed at any life stage. Rather than focusing on the consequences of a breast cancer diagnosis, women reflected on the implications of different preventative management options and the affective consequences of communicating their risk estimates to loved ones. Women also described having a good degree of control over their ability to reduce their risk and were generally knowledgeable about the early detection and preventative management options available.

**FIGURE 1:**
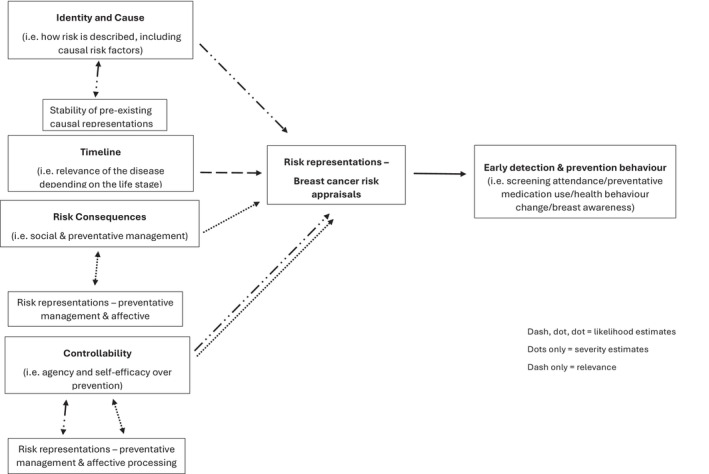
Expanded version of the IRR framework for understanding breast cancer risk representations.

Cameron's IRR framework (Cameron, 2003, 2008) largely explained women's breast cancer risk representations/appraisals following the communication of a clinically derived risk estimate, but adaptations and additions could be made to the framework to enhance its usefulness in this context (see Figure 1 for adapted IRR framework). Specifically, the findings presented here demonstrated that there was a significant overlap between how women defined breast cancer and the causes of the disease. In the IRR framework, the identity construct is defined as how individuals describe the illness, including the signs and symptoms (Cameron, 2003, 2008). As women in these studies had no first‐hand experience of the disease, the identity construct, as it is currently defined, was not particularly helpful in this context. This finding is reflected in Newby et al. (2020) research where it was suggested that the identity construct did not influence likelihood estimates when adolescents were making judgements about their CVD risk. However, the findings from the present study suggest that the identity construct when forming breast cancer risk appraisals could be redefined to encompass how women *personally identify* with the perceived causes of the illness. This self‐identification with causal risk factors seemed to significantly shape women's risk appraisals and their responses to their clinically derived risk estimates. In cases where women have not experienced breast cancer firsthand, the identity construct may be closely intertwined with causal beliefs, highlighting how personal perceptions of cause influence how risk is understood and managed. And so, the identity construct could be integrated with the causal construct in the context of understanding illness risk appraisals (see Figure 1).

As the IRR framework suggests, causal representations have a significant influence on likelihood estimates. This is in line with previous studies which have used this framework (Newby et al., 2017, 2020). For instance, the findings presented here demonstrated that women are biased towards particular risk factors, namely family history and health behaviours when forming causal representations for breast cancer, influencing likelihood estimates. These causal representations also appeared to inform pre‐existing expectations which influenced whether the clinically derived risk estimates communicated were trusted and internalized. Thus, findings from this study raise questions about the extent to which illness risk representations/ appraisals change following the communication of a clinically derived risk estimate. As the literature indicates that women's risk appraisals generally do no align with the clinical risk estimate provided (Cull et al., 1999; Lobb et al., 2003; Ozanne et al., 2010; Woof, McWilliams, et al., 2023), the IRR framework would benefit from an acknowledgment of where barriers exist in relation to the adaptation of personal risk appraisals following the communication of a clinically derived risk estimate. In this study, it has been shown that barriers appear to exist within the causal construct, as women's causal representations are incomplete and influenced by particular risk factors, affecting whether likelihood estimates change.

The IRR framework suggests that likelihood estimates are also influenced by the timeline of the disease (Cameron, 2003). Although women acknowledged that the likelihood of developing breast cancer increases with age, this fact did not appear to influence personal likelihood estimates but instead increased the relevance of the disease to women. For instance, women simply described becoming more conscious of their health with age, with breast cancers diagnosed in others of a comparable age increasing awareness. Similar findings were found in Newby et al. (2017) research, where it was described that adolescents did not feel the need to enact preventative behaviours to reduce their risk of bowel cancer at their current age. In the present study, therefore the timeline construct does not necessarily add to the understanding of women's breast cancer risk appraisals and personal evaluations of the likelihood of diagnosis.

Furthermore, the IRR framework suggests that when individuals consider the consequences of their illness risk, they appraise the severity of the illness and the treatments available, guiding their preventative behaviours (Cameron, 2003). In the present context, however, where a risk estimate had already been communicated, women focused on the consequences of the risk itself, especially if the risk was elevated. In this context, we saw affective reasoning guiding behaviour, with women identifying that the disclosure of their risk to loved ones could have negative emotional consequences. The consequences of early detection and preventative management options were also considered. Here, we saw breast cancer risk representations and severity estimates regarding preventative management options interacting. For instance, severity estimates associated with the side effects of preventative medication drove behavioural decisions not to take the medication, despite women acknowledging that medication could reduce the likelihood of developing breast cancer. Therefore, in the context of risk communication, the IRR framework would benefit from an explicit acknowledgment of risk representations associated with preventative management options and how these interact with illness risk representations to influence behaviour (see Figure 1).

Women in this study also demonstrated a good level of knowledge regarding the ways in which to control and prevent breast cancer from developing, and detailed agency and self‐efficacy in enabling them to do so. In particular, women focused on the importance of detecting breast cancer early. Here women considered how severe breast cancer could be and identified ways in which to reduce the likelihood of being diagnosed with an aggressive cancer. It was evident that women's emotional processing guided behaviour about breast screening attendance, with all women describing the reassurance it provides for every risk type. These attitudes towards screening are consistent with Waller, Osborne and Wardle's research (Waller et al., 2015) which found that 90% of 1895 respondents identified screening as ‘almost always a good idea’, with 75% also describing its value for detecting cancers early.

Additionally, it was clear in this study that the message of breast awareness and self‐examination remains prevalent, with many women remarking that checking their breasts regularly provides an opportunity to detect any changes early and reduce the likelihood of an aggressive cancer being diagnosed. Although this group of women appeared knowledgeable about this, research with younger women (aged 30–39 years) found that women lack confidence in how to perform breast checks correctly, due to their limited knowledge about what to feel and look for (Hindmarch et al., 2023). The IRR framework suggests that the controllability construct informs severity estimates (Cameron, 2003). Indeed, we have shown here that women consider the severity of breast cancer stages when guiding their preventative behaviours. However, this study has also shown that the controllability construct can also inform likelihood estimates, with women enacting preventative behaviours to reduce their likelihood of being diagnosed with an aggressive cancer (see Figure 1).

This study has shown that women often have a limited understanding of the causal factors associated with breast cancer, with family history and health behaviours dominating risk representations and appraisals. As a result, when clinical risk estimates do not align with these primary risk factors, some women question the validity of their personalized risk estimates. To address this issue, information and media campaigns should be implemented to raise awareness of lesser‐known risk factors, such as parity and age at first pregnancy. Distributing information through posters or television adverts in doctors' surgeries and breast screening sites could increase awareness and help women develop a more balanced understanding of the causes and risk factors associated with breast cancer. This approach would ensure that women receiving personalized breast cancer risk estimates are not confronted with unfamiliar information that is difficult to relate to and incorporate into their existing understandings of risk.

It was also evident in this study that representations and appraisals of preventative management options guide behaviour about their use. This was particularly obvious when women discussed the use of preventive medication, with many choosing not to take these medications despite acknowledging that their use could reduce risk. As uptake to preventative medication for breast cancer remains relatively low (Smith et al., 2016), HCPs should consider how risk representations, risk factor knowledge and representations about preventative management interact to inform behaviour and identify areas where concerns could be alleviated. For instance, questions tailored to align with the constructs of the IRR framework could be incorporated into clinical practice to identify areas where women may have misunderstandings, gaps in knowledge or concerns. For example, when eliciting views regarding preventative management, HCPs may wish to draw on the consequences and controllability constructs of the IRR framework by posing questions such as ‘Do you think that taking preventative steps now will reduce the chances of a serious breast cancer diagnosis in the future?’ or ‘Do you feel that you have control over whether or not you get breast cancer?’. By working through the answers to such questions, HCPs could provide more personalized consultations, addressing concerns and knowledge gaps, thus supporting informed decision‐making about preventative care.

To our knowledge, this study is the first to provide an understanding of the ideas and beliefs that underlie women's breast cancer risk representations and appraisals following notification of a clinically derived risk estimate in a breast screening programme. The framework analysis in this study utilized both deductive and inductive approaches. Deductive coding was guided by the IRR framework, while inductive coding identified aspects of women's breast cancer risk representations and appraisals that were not covered by the original IRR framework. Combining both approaches led to a more robust and nuanced understanding of the data by creating a balance between testing the utility of the IRR framework (deductive) and developing new insights (inductive).

However, the study is not without its limitations. As this was a secondary analysis of existing qualitative data, it is important to situate the findings of this study in the context from which the data originated. For instance, the topic guides used for the two studies presented here were not developed to assess the utility of the IRR framework. This meant that some of the data, especially the data collected in study two (McWilliams et al., 2023) did not align with the aims of the present study. This meant that large amounts of data from these studies were not applicable. Furthermore, when conducting the primary analysis for study one (Woof, McWilliams, et al., 2023), it was clear to the primary author that features of the IRR framework were present. The data from study one (Woof, McWilliams, et al., 2023) were therefore used to assess the IRR framework; however, due to the limited number of interviews, it was deemed important to analyse this data together with additional data from the BC‐Predict study, where data on breast cancer risk appraisals were also present.

Throughout the secondary analysis process, it was crucial for the primary author to remain aware that the data from study one (Woof, McWilliams, et al., 2023) aligned more closely with the current research question, ensuring that this dataset was not given undue weight in the analysis. This was particularly important as the primary author had collected and analysed the data in study one (Woof, McWilliams, et al., 2023), while only serving in an advisory capacity for study two (McWilliams et al., 2023). However, the analysis revealed that despite the interviews in study two (McWilliams et al., 2023) not being designed with this goal in mind, the data provided evidence supporting the IRR framework. This, in turn, reinforced the findings from study one (Woof, McWilliams, et al., 2023) and highlighted the IRR framework's relevance in understanding responses to clinically derived breast cancer risk estimates. Nonetheless, the primary author remained mindful of their deeper familiarity with the data from study one (Woof, McWilliams, et al., 2023), recognizing that without careful consideration, this could have led to a biased analysis.

Finally, the sample of women from which the data originated were predominately White British, living in areas of low deprivation based on their postcode data. It is therefore uncertain how well the IRR framework would work at explaining breast cancer risk representations and appraisals in other groups of women.

Since this study conducted a secondary analysis of existing qualitative data, further research focusing primarily on assessing the utility of the IRR framework would be beneficial, utilizing a topic guide designed for this purpose. With certain adaptations, this study has demonstrated that the IRR framework is effective in understanding women's breast cancer risk representations and appraisals. In particular, the present analysis has demonstrated that adaptations to the framework in terms of an appreciation of whether pre‐existing risk representations/appraisals change following communication of a risk estimate is needed. Future research should consider how useful this adapted framework is in a sample where risk appraisals have been found to change (Woof et al., 2024; Woof, Howell, et al., 2023). In addition, future research should also evaluate the framework's applicability in other disease areas where risk estimates are provided, such as CVD and diabetes. Furthermore, since the women in this study were predominantly White British and middle class (based on postcode data), further research should evaluate the utility of the IRR framework in a more ethnically and socially diverse sample.

## CONCLUSION

In summary, this study has demonstrated that women have a good understanding of breast cancer prevention and early detection measures. However, there were gaps in their knowledge regarding the causes and risk factors associated with breast cancer, affecting their perceptions of their personal risk. Cameron's IRR framework (Cameron, 2003, 2008) was found to largely explain women's breast cancer risk representations and appraisals, but adaptations were suggested to improve its applicability. For example, redefining and amalgamating the identity construct with the causal construct and having a more explicit focus on the impact of representations of preventative management options on behaviour. It was also argued that the framework would benefit from understanding how and whether pre‐existing risk representations/ appraisals change after receiving clinically derived risk estimates. Finally, HCPs should assess women's knowledge of breast cancer risk factors and causes before providing personal risk estimates to reduce confusion and uncertainty.

## AUTHOR CONTRIBUTIONS

**Victoria G. Woof:** Conceptualization; methodology; formal analysis; investigation; funding acquisition; writing – original draft; writing – review and editing; data curation. **Lorna McWilliams:** Conceptualization; methodology; data curation; supervision; writing – review and editing; funding acquisition. **D. Gareth Evans:** Conceptualization; methodology; supervision; writing – review and editing; funding acquisition. **Anthony Howell:** Conceptualization; methodology; writing – review and editing; supervision; funding acquisition. **David P. French:** Supervision; conceptualization; methodology; formal analysis; funding acquisition; writing – review and editing.

## CONFLICT OF INTEREST STATEMENT

The authors affirm that there are no potential conflicts of interest regarding the research, authorship and publication of this article.

## ETHICS STATEMENT

Ethical approval for Woof, McWilliams, et al. (2023) and McWilliams et al. (2023) where the data for this secondary analysis originated was granted by the North West—Greater Manchester East Research Ethics Committee (18/NW/0856). All participants provided verbal informed consent which was audio‐recorded.

## Supporting information


Data S1:

## Data Availability

The datasets used for analysis in this study are not publicly accessible due to potential privacy implications that could compromise participant consent. Data related to the analysis process can be found on Figshare (add link). Please contact the corresponding author for further details regarding data availability.

## References

[bjhp12792-bib-0001] Bayne, M. , Fairey, M. , Silarova, B. , Griffin, S. J. , Sharp, S. J. , Klein, W. M. , Sutton, S. , & Usher‐Smith, J. A. (2020). Effect of interventions including provision of personalised cancer risk information on accuracy of risk perception and psychological responses: a systematic review and meta‐analysis. Patient Education and Counseling, 103(1), 83–95. 31439435 10.1016/j.pec.2019.08.010 PMC6919334

[bjhp12792-bib-0002] Brooks, J. , & King, N. (2017). Approaches to qualitative psychology. Applied Qualitative Research in Psychology, 26, 14–32.

[bjhp12792-bib-0003] Cameron, L. D. (2003). Conceptualizing and assessing risk perceptions: A self‐regulatory perspective. In National Cancer Institute workshop on conceptualizing and measuring risk perception (pp. 13–14).

[bjhp12792-bib-0004] Cameron, L. D. (2008). Illness risk representations and motivations to engage in protective behavior: The case of skin cancer risk. Psychology & Health, 23(1), 91–112. 25159909 10.1080/14768320701342383

[bjhp12792-bib-0005] Cull, A. , Anderson, E. D. , Campbell, S. , Mackay, J. , Smyth, E. , & Steel, M. (1999). The impact of genetic counselling about breast cancer risk on women's risk perceptions and levels of distress. British Journal of Cancer, 79(3), 501–508. 10027320 10.1038/sj.bjc.6690078 PMC2362435

[bjhp12792-bib-0006] Evans, D. G. , McWilliams, L. , Astley, S. , Brentnall, A. R. , Cuzick, J. , Dobrashian, R. , Duffy, S. W. , Gorman, L. S. , Harkness, E. F. , Harrison, F. , & Harvie, M. (2023). Quantifying the effects of risk‐stratified breast cancer screening when delivered in real time as routine practice versus usual screening: The BC‐predict non‐randomised controlled study (NCT04359420). British Journal of Cancer, 128(11), 2063–2071. 37005486 10.1038/s41416-023-02250-w PMC10066938

[bjhp12792-bib-0007] French, D. P. , Astley, S. , Brentnall, A. R. , Cuzick, J. , Dobrashian, R. , Duffy, S. W. , Gorman, L. S. , Harkness, E. F. , Harrison, F. , Harvie, M. , & Howell, A. (2020). What are the benefits and harms of risk stratified screening as part of the NHS breast screening Programme? Study protocol for a multi‐site non‐randomised comparison of BC‐predict versus usual screening (NCT04359420). BMC Cancer, 20, 1–4. 10.1186/s12885-020-07054-2 PMC7302349 32552763

[bjhp12792-bib-0008] Gale, N. K. , Heath, G. , Cameron, E. , Rashid, S. , & Redwood, S. (2013). Using the framework method for the analysis of qualitative data in multi‐disciplinary health research. BMC Medical Research Methodology, 13, 1–8. 24047204 10.1186/1471-2288-13-117 PMC3848812

[bjhp12792-bib-0009] Heaton, J. (2008). Secondary analysis of qualitative data. In The SAGE Handbook of Social Research Methods (pp. 506–519). SAGE Publications Ltd.

[bjhp12792-bib-0010] Hindmarch, S. , Gorman, L. , Hawkes, R. E. , Howell, S. J. , & French, D. P. (2023). “I don't know what I'm feeling for”: Young women's beliefs about breast cancer risk and experiences of breast awareness. BMC Women's Health, 23(1), 312. 37328760 10.1186/s12905-023-02441-w PMC10276361

[bjhp12792-bib-0011] Leventhal, H. , Benyamini, Y. , Brownlee, S. , Diefenbach, M. , Leventhal, E. A. , Patrick‐Miller, L. , & Robitaille, C. (2013). Illness representations: Theoretical foundations. In Perceptions of health and illness (pp. 19–45). Psychology Press.

[bjhp12792-bib-0012] Leventhal, H. , Brissette, I. , & Leventhal, E. A. (2012). The common‐sense model of self‐regulation of health and illness. In The self‐regulation of health and illness behaviour (pp. 43–66). Routledge.

[bjhp12792-bib-0013] Lobb, E. A. , Butow, P. N. , Meiser, B. , Barratt, A. , Gaff, C. , Young, M. A. , Kirk, J. , Gattas, M. , Gleeson, M. , & Tucker, K. (2003). Women's preferences and consultants' communication of risk in consultations about familial breast cancer: Impact on patient outcomes. Journal of Medical Genetics, 40(5), e56. 12746410 10.1136/jmg.40.5.e56 PMC1735473

[bjhp12792-bib-0014] McWilliams, L. , Evans, D. G. , Payne, K. , Harrison, F. , Howell, A. , Howell, S. J. , French, D. P. , & Breast Screening Risk‐Stratification Agenda Setting Group . (2022). Implementing risk‐stratified breast screening in England: An agenda setting meeting. Cancers, 14, 4636. 10.3390/cancers14194636 36230559 PMC9563640

[bjhp12792-bib-0015] McWilliams, L. , Ruane, H. , Ulph, F. , Woof, V. G. , Harrison, F. , Evans, D. G. , & French, D. P. (2023). What do women think about having received their breast cancer risk as part of a risk‐stratified NHS breast screening Programme? A qualitative study. British Journal of Cancer, 129(2), 356–365. 37225893 10.1038/s41416-023-02268-0 PMC10206350

[bjhp12792-bib-0016] Ministry of Housing, Communities, Local Government . (2019). English indices of deprivation 2019. https://www.gov.uk/government/statistics/english‐indices‐of‐deprivation‐2019

[bjhp12792-bib-0017] National Institute for Health and Care Excellence (NICE) . (2013). Familial breast cancer: Classification, care and managing breast cancer and related risks in people with a family history of breast cancer. National Institute for Health and Care Excellence. https://www.nice.org.uk/guidance/cg164/resources/familial‐breast‐cancer‐classification‐care‐and‐managing‐breast‐cancer‐and‐related‐risks‐in‐people‐with‐a‐family‐history‐of‐breast‐cancer‐pdf‐35109691767493 31940157

[bjhp12792-bib-0018] Newby, K. , Varnes, L. , Yorke, E. , Meisel, S. F. , & Fisher, A. (2020). Illness risk representation beliefs underlying adolescents' cardiovascular disease risk appraisals and the preventative role of physical activity. British Journal of Health Psychology, 25(1), 171–188. 31814243 10.1111/bjhp.12400

[bjhp12792-bib-0019] Newby, K. V. , Cook, C. , Meisel, S. F. , Webb, T. L. , Fisher, B. , & Fisher, A. (2017). Young people's beliefs about the risk of bowel cancer and its link with physical activity. British Journal of Health Psychology, 22(3), 449–462. 28419714 10.1111/bjhp.12238

[bjhp12792-bib-0020] Ozanne, E. M. , Wittenberg, E. , Garber, J. E. , & Weeks, J. C. (2010). Breast cancer prevention: Patient decision making and risk communication in the high risk setting. The Breast Journal, 16(1), 38–47. 19889168 10.1111/j.1524-4741.2009.00857.x

[bjhp12792-bib-0021] Ritchie, J. , & Spencer, L. (2002). Qualitative data analysis for applied policy research. In Analyzing qualitative data (pp. 173–194). Routledge.

[bjhp12792-bib-0022] Ruggiano, N. , & Perry, T. E. (2019). Conducting secondary analysis of qualitative data: Should we, can we, and how? Qualitative Social Work, 18(1), 81–97. 30906228 10.1177/1473325017700701 PMC6428200

[bjhp12792-bib-0023] Smith, S. G. , Sestak, I. , Forster, A. , Partridge, A. , Side, L. , Wolf, M. S. , Horne, R. , Wardle, J. , & Cuzick, J. (2016). Factors affecting uptake and adherence to breast cancer chemoprevention: A systematic review and meta‐analysis. Annals of Oncology, 27(4), 575–590. 26646754 10.1093/annonc/mdv590 PMC4803450

[bjhp12792-bib-0024] Waller, J. , Osborne, K. , & Wardle, J. (2015). Enthusiasm for cancer screening in Great Britain: A general population survey. British Journal of Cancer, 112(3), 562–566. 25535731 10.1038/bjc.2014.643 PMC4453657

[bjhp12792-bib-0025] Woof, V. G. , Howell, A. , Fox, L. , McWilliams, L. , Evans, D. G. , & French, D. P. (2023). How do women experience a change in their clinically‐derived breast cancer risk estimates: Views from a UK family history risk and prevention clinic. 10.21203/rs.3.rs-3643438/v1 PMC7616752 39356295

[bjhp12792-bib-0026] Woof, V. G. , Howell, A. , Fox, L. , McWilliams, L. , Evans, D. G. , & French, D. P. (2024). Are women's breast cancer risk appraisals in line with updated clinical risk estimates communicated? Results from a UK family history risk and prevention clinic. Cancer Epidemiology, Biomarkers & Prevention, 33(12), 1671–1677. 10.1158/1055-9965.EPI-24-0581 PMC7616752 39356295

[bjhp12792-bib-0027] Woof, V. G. , Howell, A. , McWilliams, L. , Gareth Evans, D. , & French, D. P. (2022). How do women who are informed that they are at increased risk of breast cancer appraise their risk? A systematic review of qualitative research. British Journal of Cancer, 127(11), 1916–1924. 36002751 10.1038/s41416-022-01944-x PMC9681857

[bjhp12792-bib-0028] Woof, V. G. , McWilliams, L. , Howell, A. , Evans, D. G. , & French, D. P. (2023). How do women at increased risk of breast cancer make sense of their risk? An interpretative phenomenological analysis. British Journal of Health Psychology, 28(4), 1169–1184. 37395149 10.1111/bjhp.12678 PMC10947456

